# WMSS: A Web-Based Multitiered Surveillance System for Predicting CLABSI

**DOI:** 10.1155/2018/5419313

**Published:** 2018-07-05

**Authors:** Amin Y. Noaman, Abdul Hamid M. Ragab, Nabeela Al-Abdullah, Arwa Jamjoom, Farrukh Nadeem, Anser G. Ali

**Affiliations:** ^1^Department of Computer Science, Faculty of Computing and Information Technology, King Abdulaziz University, Jeddah, Saudi Arabia; ^2^Department of Information Systems, Faculty of Computing and Information Technology, King Abdulaziz University, Jeddah, Saudi Arabia; ^3^Clinical Epidemiology & Infection Control, Faculty of Nursing, King Abdulaziz University, Jeddah, Saudi Arabia

## Abstract

Central-line-associated bloodstream infection (CLABSI) rates are a key quality metric for comparing hospital quality and safety. Manual surveillance systems for CLABSIs are time-consuming and often limited to intensive care units (ICUs). A computer-automated method of CLABSI detection can improve the validity of surveillance. A new web-based, multitiered surveillance system for predicting and reducing CLABSI is proposed. The system has the capability to collect patient-related data from hospital databases and hence predict the patient infection automatically based on knowledge discovery rules and CLABSI decision standard algorithms. In addition, the system has a built-in simulator for generating patients' data records, when needed, offering the capability to train nurses and medical staff for enhancing their qualifications. Applying the proposed system, both CLABSI rates and patient treatment costs can be reduced significantly. The system has many benefits, among which there is the following: it is a web-based system that can collect real patients' data from many IT resources using iPhone, iPad, laptops, Internet, scanners, and hospital databases. These facilities help to collect patients' actual data quickly and safely in electronic format and hence predict CLABSI efficiently. Automation of the patients' data diagnosis process helps in reducing CLABSI detection times. The system is multimedia-based; it uses text, colors, and graphics to enhance patient healthcare report generation and charts. It helps healthcare decision makers to review and approve policies and surveillance plans to reduce and prevent CLABSI.

## 1. Introduction

Hospital-acquired infections (HAIs), also known as nosocomial infections, are caused by viral, bacterial, and fungal pathogens; the most common types are bloodstream infection (BSI), ventilator-associated pneumonia (VAP), urinary tract infection (UTI), and surgical site infection (SSI), as explained by K. Woeltje et al. [1]. Such infections can be acquired in hospital or in other clinical settings. The infection may be spread by various means to the susceptible patient in the clinical settings. These means may include infected staff, infected patients, contaminated equipment, bed linen, or air droplets. The source of the infection may also come from the outside environment, or from other sources that cannot be determined, or after a surgery that compromises the protective skin barrier. The Centers for Disease Control and Prevention (CDC) estimated roughly 1.7 million hospital-associated infections in the United States from all types of microorganisms. These included bacteria and fungi combined, causing about ninety-nine thousand deaths each year as described by Haidee and Russell [2].

In Europe, where hospital surveys have been conducted, “gram-negative infections are estimated to account for two-thirds of the 25,000 deaths each year” [3]. Nosocomial infections can cause severe pneumonia and infections of the urinary tract and bloodstream. Many types of nosocomial infections are hard to treat with antibiotics. In addition, antibiotic resistance can complicate treatment.

Central Line Associated Bloodstream Infections (CLABSI) are serious infections typically causing a prolongation of hospital stay and increased cost and risk of mortality. However, “CLABSI can be prevented through proper insertion techniques and management of the central line”. These techniques are addressed by the CDC's Healthcare Infection Control Practices Advisory Committee (CDC/HIPAC) [4].

This paper describes a web-based, multitiered surveillance system for predicting and reducing CLABSI. The word “multitiered” means that the system can work using actual patients' real data collected from the web-resources and from hospitals databases. It can also work using patients' data records that can be generated for training by the built-in system simulator; i.e., the system works in two levels of data (actual data and the simulated data). CLABSI is “a primary laboratory confirmed bloodstream infection in a patient with a central line at the time of (or within 48-hours prior to) the onset of symptoms and the infection is not related to an infection from another site” [3]. The blood cultures must be taken from the central line and simultaneously from a peripheral vein, with a shorter time to positivity in the central line sample. Several researchers have tackled the problem of detecting and preventing CLABSI hospital-acquired infections. However, in developed countries it is difficult to get patient data available in electronic formats suitable for use in computing facilities. Even most of the available patients' data in the hospitals are written in paper format and are mostly confidential. To overcome these problems, we have built a simulator to generate CLABSI patients' data records to be able to build and test our proposed system; in addition, it transforms a patient's paper records into an electronic format. This proved very helpful as a system for training healthcare staff members and nurses. The system uses multimedia so that diagnosis reports can be produced in the form of text tables as well as in the form of colored graphics and charts.

The rest of the paper covers the following: Section 2 discusses types of surveillance systems and comparison.  Section 3 describes materials and methods.  Section 4 explains system results and discussions, and Section 5 is the conclusion.

## 2. Surveillance Systems Comparison

Healthcare-associated infections (HAIs) are a major global public health concern. In developing countries, the lack of surveillance systems leads to an underestimation of the global burden of HAI as explained by Maha Talaat et al. [7]. CLABSIs still occur in intensive care units in developed countries (high income countries) in the US and Europe [4] and in the developing countries (middle/low income countries) in Asia and Africa. Intensive care units (ICUs) in countries with limited resources “have rates of device-associated healthcare-associated infection including CLABSI, VAP, and catheter-associated urinary tract infection (CAUTI) that are 3 to 5 times higher than rates reported from North American, Western European, and Australian ICUs”, as explained by Victor D. Rosenthal et al. [8].

Although computer algorithms to detect bloodstream infections can be used to reduce CLABSI, as described by Trick et al. [9], they stated that “automated bloodstream infection surveillance with electronic data is an accurate alternative to surveillance with manually collected data”. Also, a comparison of automated strategies for surveillance of nosocomial bacteremia was investigated by Cristina Bellini et al. [10]. They concluded that automated strategies are convenient alternatives to manual surveillance of nosocomial BSI. An automated CLABSI surveillance approach has also several advantages, including reducing the time spent by infection control specialists on routine surveillance and reallocating it to prevention efforts and potentially extending surveillance outside of the intensive care unit (ICU) [11].

Automated algorithmic approaches for translating manual infection control surveillance methods were explained by Bala Hota et al. [12]. The goal was to automat infection detection that would achieve the CDC aim and to improve the financial support for hospitals to deploy electronic health records.

A semiautomated electronic surveillance system for ventilator-associated pneumonia (VAP) and CLABSI in a Dutch intensive care unit was described by Anna Maria Kaiser [13]. They concluded that surveillance of VAP and CLABSI with a trigger-based electronic surveillance system (ESS) is feasible and effective because of its time-saving benefits. An automated surveillance system was described by Keith F. Woeltje [14]; it uses existing databases with patient-level variables and microbiology data. It can perform acceptably good surveillance in areas where resources do not allow for traditional manual surveillance.

Due to the unavailability of patient data and confidentiality issues, specifically in developing countries simulated patient records can be used. Simulation-based training associated with reduced CLABSI rates was used by Gilman B. Allen [15]. In their medical and surgical intensive care units, they obtained the benefits of simulation training, electronic medical records, and standardized kits to reduce their CLABSI rates. The several benefits of CLABSI simulation training were explained by Karla M. Abela [16], among which there is the following: learning environment is safe. Teaching methodology is flexible. Patient safety is not compromised. Feedbacked data can be structured immediately. Student focused learning may be individualized. Simulation-based training can significantly improve nurses' education quality and hence leads to reduced CLABSI rates as explained by Khouli H et al. [17] and by Barsuk JH. et al. [18]. Also, simulation training can produce significant medical care cost savings, as explained by Elaine R. Cohen [19].

## 3. Materials and Methods

This section describes the architecture of the proposed system, including materials used and methods.

### 3.1. System Hardware Components

**Figure 1:**
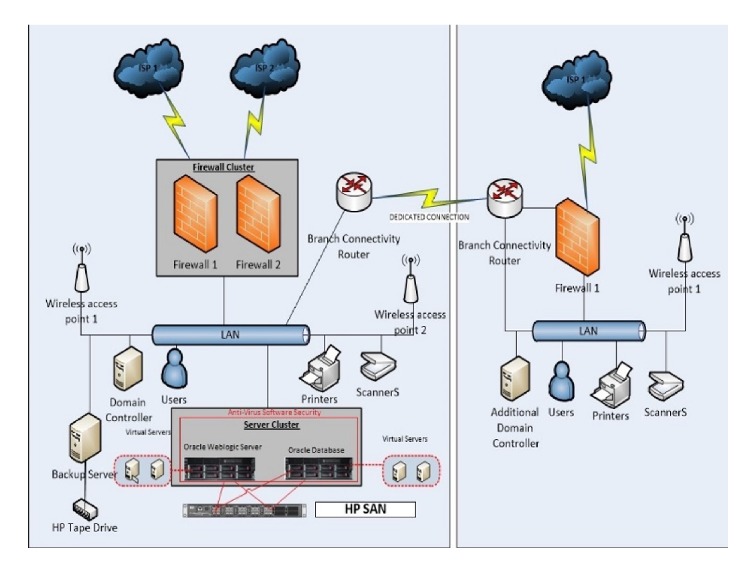
The system hardware web components developed.

Figure 1 shows the system hardware web components developed, based on oracle architecture. To ensure system data protection activity and to ensure security and safety, the following steps has been implemented:

1. Users are connected to the Hospital Servers through firewalls.
2. Users are connected to fill data through a secure Java Web start application maintained by Oracle Web-Logic Server.
3. Data and application stores in Server Cluster are protected using Antivirus Software Security.
4. Also, the data is secured by Default Oracle Database Security.
5. The Clinical Infections Department at King Abdulaziz University Hospital was connected to the system during the experimental stage; it covers 750 beds.
6. Several local hospitals are connected to the system during the experimental stage.

### 3.2. System Software Components

**Figure 2:**
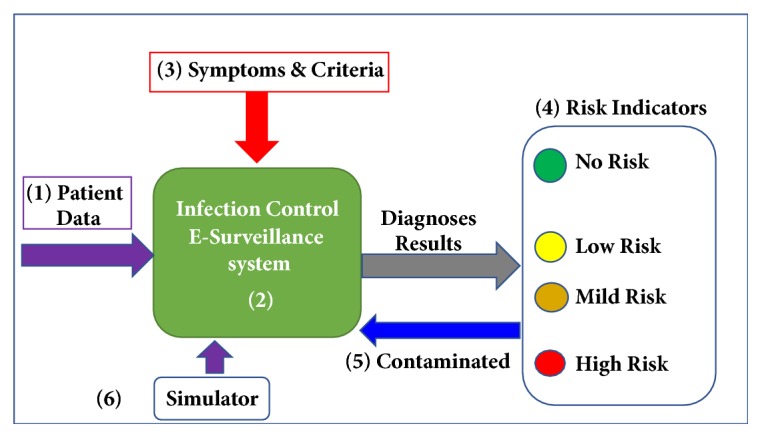
The proposed WMSS-CLABSI system software components.

The proposed system software is constructed of six components (modules), as shown in Figure 2. These include the Patients' Data Module (PDM), the CLABSI algorithm Module (CAM), the Symptoms and Criteria Module (SCM), the Risk Indicators Module (RIM), the Contamination Module (CM), and the Simulator module (SM). The functions of these components are described in the following sections, respectively.

#### 3.2.1. Patients' Data Module (PDM)

This software component is responsible for collecting patients' data from several resources from hospitals locally or remotely from the portal web site. This can be done using many IT resources including iPhones, iPad, laptops, the Internet, scanners, and hospital databases.  Table 1 shows a part of the primary bloodstream infections entry form and instructions for completion [20]. To detect if a patient has a CLABSI infection or not, additional patient data including symptoms criteria is inputted to the surveillance system proposed, as explained in the next sections.

#### 3.2.2. CLABSI Algorithm Module (CAM)

This system software component is responsible for applying the standard CLABSI algorithm as shown in Figure 2. It is used for diagnosis patients' data collected. A positive blood culture with signs and symptoms of infection are evidence of a bloodstream infection. A patient may develop a CLABSI case, when

1. s/he suffers from fevers, chills, or the skin around the catheter may become sore and red.
2. When a bloodstream infection occurs and there is no other place where that infection could be coming from.
3. The infection is associated with the central line.
4. It is still possible that the infection could be coming from another place but that source is hidden [5].

The CLABSI algorithm deals with these symptoms to know and detect the type of infection, as explained in Section 3.2.3.

#### 3.2.3. Symptoms and Criteria Module (SCM)

This system software component is responsible for finding out CLABSI symptoms and evaluating their criteria, for diagnosis of patients' data. We built the list of CLABSI symptoms and criteria based on hospital-acquired infection definition described in [5, 20]. In our experimental stage, we have applied twenty-four CLABSI predictors that are shown in Table 2. They can be used effectively for predicting patients' infections.

#### 3.2.4. Risk Indicators Module (RIM)

This system software component is utilized to estimate patient illness due to predicted infection, as shown in Table 3.

The risk indicator software module computes total symptoms weights and indicates CLABSI prediction results, in the form of colors, as shown in Table 3.  Table 4 shows CLABSI predictors and symptoms in detail and the VALUE-LABLE risk weights (high, mild, low, and no risk) used for infection prediction. Four levels of infection indicators (A, B, C, and D) are proposed. Red color indicates CLABSI high risk (A), when sum of high symptoms criteria weights was more than or equal five. Orange color indicates CLABSI mild risk (B) when sum of mild symptoms criteria weights was more than or equal five. Yellow color indicates CLABSI low risk (C) when sum of low symptoms criteria weights was more than or equal five. Green color indicates no risk (D) when sum of normal symptoms criteria weights was more than or equal five. At least the sum of a total of five symptoms criteria should be achieved to justify an infected prediction for CLABSI patient cases.

Using of color indicators are an appropriate classification of the patients' risk of developing CLABSI, because it is based on the electronic hospital-acquired infection surveillance Phoenix system used at KAUH, which uses colors. And we established it based on other classification, such as ASA (American Society of Anesthesiologists) physical status classification system [21]. Also, the color indicators are suitable for providing a quick vision test, for indicator that can be figured out by staff members and nurses, specifically suitable in case of training. In addition, the system can also produce detailed results of patients' diagnosis data in the form of tables and charts, as explained in next sections.

#### 3.2.5. Contamination Module (CM)

This system software component is responsible for* producing feedback information* after a diagnosis of a CLABSI patient case. It tells the nurses and medical staff whether the patient is now infected or not. If a patient is infected, patient treatment by staff and nurses should be carried out quickly to cure the patient safely. Then, the system continues to remeasure the patient case, until s/he is recovered from the infection.

#### 3.2.6. Simulator Module (SM)

This system software component was built to simulate patient data records automatically, when required for training. It is used for testing the system's CLABSI prediction and patient data validation. In addition, it is used for nurses' education and medical staff training. To proof that the simulator generates patients' data records and gives almost identical results as the actual patients' data records, the following steps are implemented:

1. The simulator generates patient prediction records randomly. These records are generated by Oracle database procedure.
2. The prediction values are selected from a predefined set of values. These procedure uses the default Oracle random procedure.
3. The interdependencies of predictors have been implemented (like birth weight predictor links with newborn predictor, parenteral nutrition links with gender or multiple insertion with status of procedure).
4. By default, the procedure generates 10 patients' records. To proof that simulation results are equivalent and almost identical into real results, we apply the same algorithms and formulas used in the case of actual data.
5. The simulator algorithm checks if the predictor's values are valid as the actual values, then results will be accepted.

**Figure 3:**
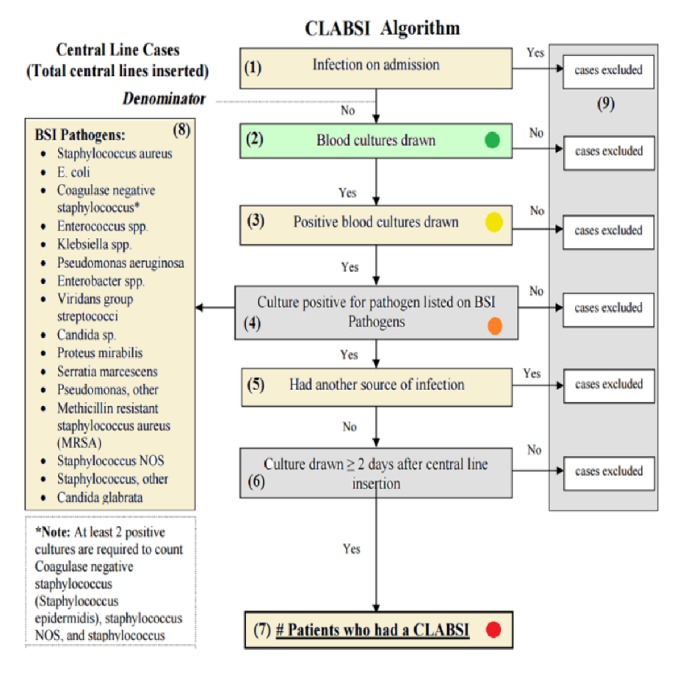
The standard CLABSI algorithm adapted from [5, 6].

Figure 3, for example, shows a sample of a simulator generated patient records. When comparing this simulated patient data records with the actual real data records, as shown in Figure 5, it indicates that the results obtained are identical.

## 4. Results and Discussions

### 4.1. Patient Information Input Screen

**Figure 4:**
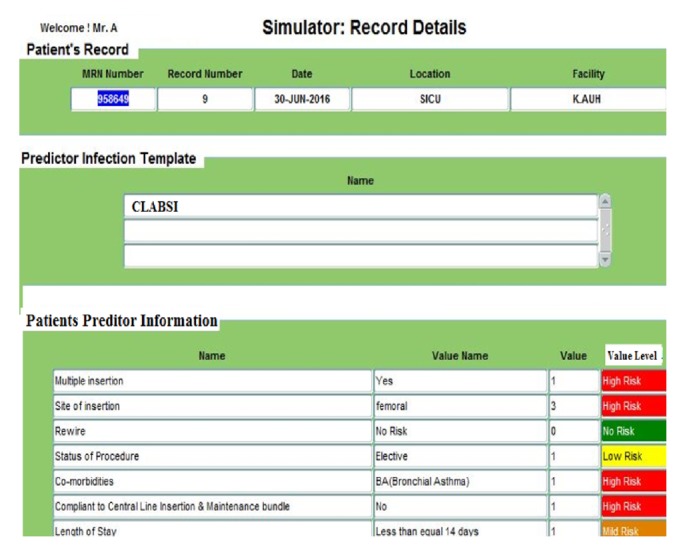
A sample of a simulator generated patient record.

**Figure 5:**
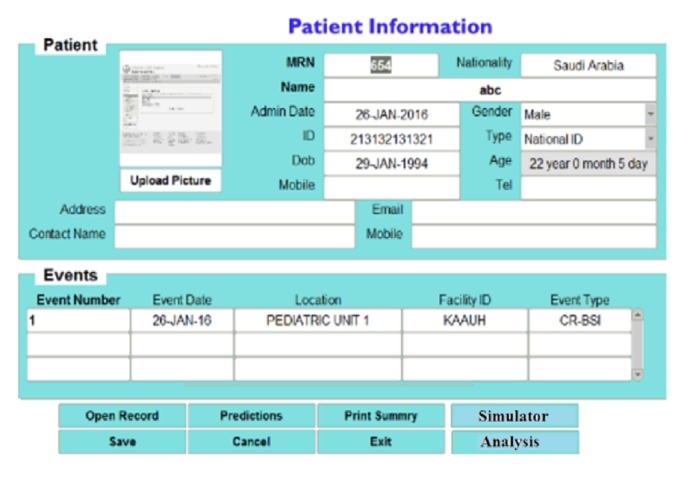
Patient information data entry form.

**Figure 6:**
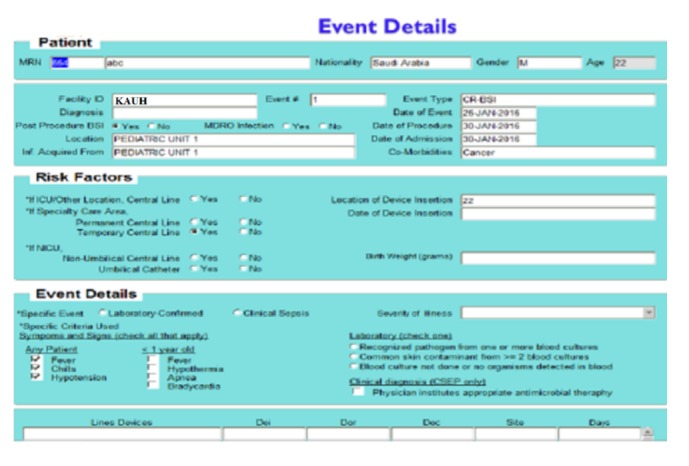
Patient event details data entry form.

**Figure 7:**
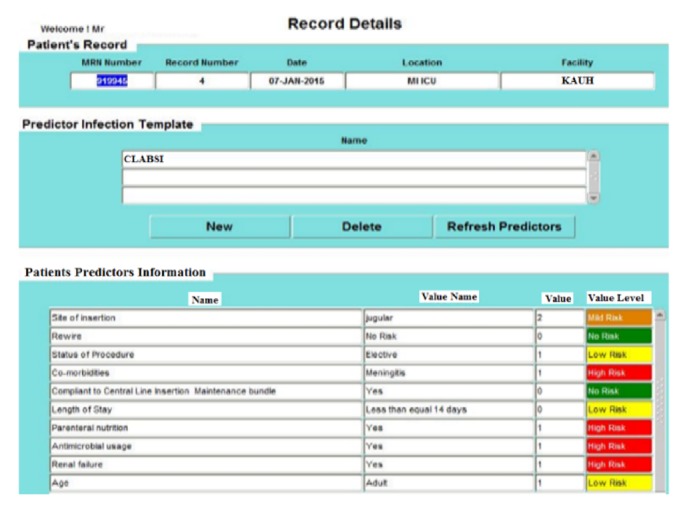
Sample of patient record details and predictors information.

The results explained here are based on real patients' data from King Abdulaziz University Hospital (KAUH), containing 750 beds, dept. of clinical infection control.  Figure 4 shows the patient information input screen. Figure 5 shows the event details input screen. The built-in simulator is an additional facility for generating artificial patient data records that can be used when needed by staff and nurses to input simulated patient data records for training and education. The proposed WMSS-CLABSI system produces several types of charts, graphs, tables, and reports of patients' information, including infection detection and diagnosis.  Figure 6 shows patient event details of the data entry form.  Figure 7 shows a sample of patient record details and patient predictors information.

### 4.2. Reports Generated and Risk Prediction

**Figure 8:**
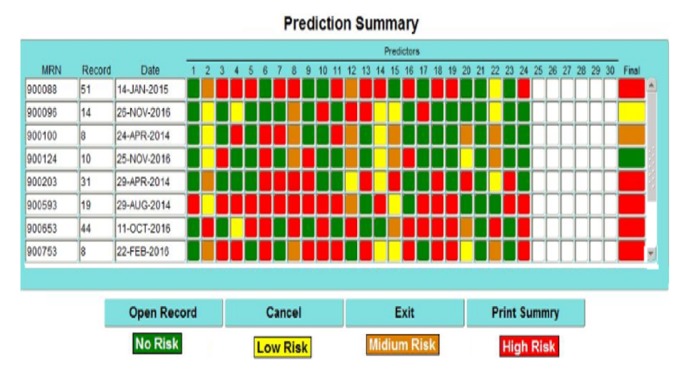
A sample of the generated prediction summary report.

Figure 8 shows a sample of the generated prediction summary report. It indicates patient's number of records, event dates, and symptoms with diagnosis indicators. Based on these patients' information, the percentage of infections risk can be computed as indicated in Table 5. It shows the theoretically percentage of types of CLABSI risk for each group (defined in Table 4) including high, mild, low, and normal risks. Each cell value in Table 5 is computed by dividing predicted numbers of CLABSI risk types by total symptoms (24) multiplied in 100.

### 4.3. Device Utilization and Standard Infection Ratios

**Figure 9:**
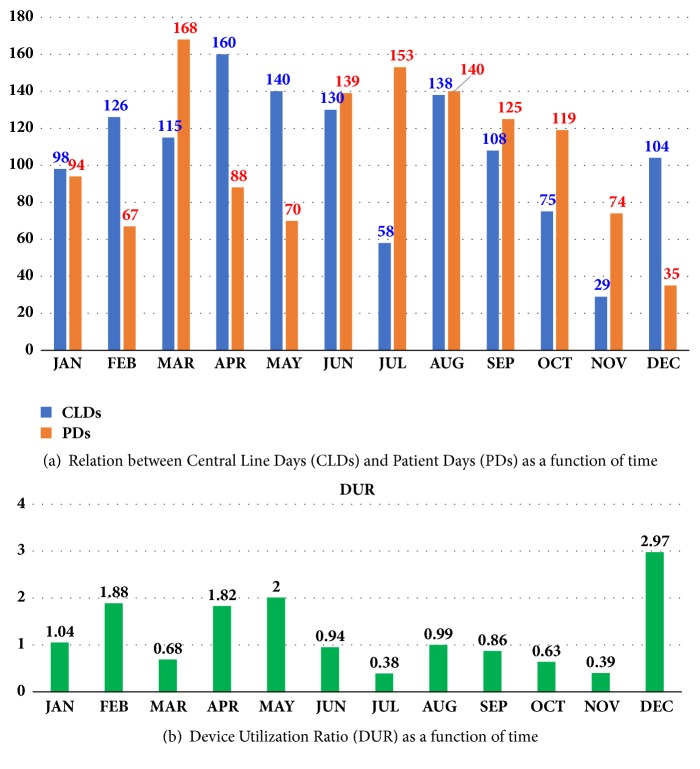


Figures 9(a) and 9(b) show a bar graph chart indicating the relationship between Central Line Days (CLDs), Patient Days (PDs), and Device Utilization Ratio (DUR) as a function of time (in months). For example, it shows that, during July, the CLDs=85 and PDs=153, then DUR = 85 / 153 = 0.38.

**Figure 10:**
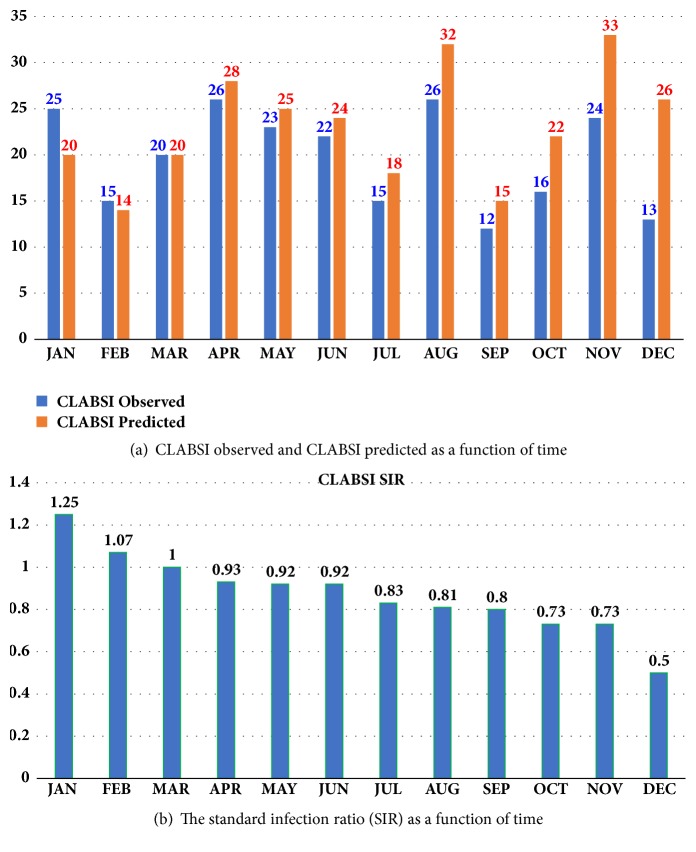


Figures 10(a) and 10(b) show some results using bar chart graphs for computing the standard infection ratio (SIR) as a function of time. SIR is computed by dividing CLABSI observed cases over CLABSI predicted cases. It is one of the important measures for judging the quality of a hospital's CLABSI detection and prevention. The standard value of SIR is one. When SIR has a value below one it indicates better hospital safety and vice versa. The higher ratio of SIR reflects a need for stronger CLABSI prevention efforts, while the lower SIR ratio reflects a robust CLABSI prevention strategies [22].

Figure 10(b) shows that there is a regularly smooth reduction in SIR ratio, as function of time (in months). This is a good indication factor. The average SIR ratio within the 1^st^ six months (from Jan. up to Jun.) is 0.86, based on CLABSI traditional manual method used. And during the next six months (from Jul. up to Dec.), the average SIR ratio within these six months is 0.73, where the automated proposed method was used. These results give a reduction in the SIR ratio = 86-73=13%. Hence, the system improvement value = 87%. This proves that the proposed automated system gives improvement in CLABSI prediction performance better than the traditional manual method. Hence, based on these results, applying the proposed method achieves better regular reduction in SIR, as function of time in months, and better than that achieved by the manual method.

## 5. Conclusions

This paper presented a new web-based, multitiered surveillance system for predicting central line associated bloodstream infections (CLABSI). The system collects patients' data from several resources including laptops, Internet, scanners, and hospitals databases. This process helps hospitals to gather patients' data efficiently and accurately. Patients' records are automatically processed for CLABSI diagnoses and prediction based on knowledge discovery CLABSI algorithms. This helps to reduce the process of infection prediction time and hence reduce CLABSIs rates. The outputs of predicted infected cases are produced using visualized multimedia forms including text reports, colored risk indicators, and graphic charts. This helps medical decision makers to review and approve policies for surveillance plans. The system has also a built-in simulator to simulate specific patient data records, when needed, for nurses' training and education. This helps in reducing and preventing many CLABSIs cases and hence keeps a hospital safe while reducing treatment costs. We are currently expanding the WMSS-CLABSI system to include ventilator-associated pneumonia (VAP), urinary tract infection (UTI), and surgical site infection (SSI), at King Abdulaziz University Hospital, in Jeddah, Saudi Arabia.

## Figures and Tables

**Table 1:** Primary bloodstream infection (BSI) form and instructions for completion (CDC 57.108) [3].

Data Field	Instructions for Data Collection
Facility ID	It will be auto entered by the computer.

Event #	It will be auto entered by the computer.

Patient ID	Required. Enter the alphanumeric patient ID number. It consists of any combination of numbers and/or letters.

Social Security #	Optional. Enter the 9-digit numeric patient Social Security Number.

Secondary ID	Optional. Enter the alphanumeric ID number assigned by the facility.

Medicare #	Conditionally required. Enter the patient's Medicare number for all events reported as part of a CMS Quality Reporting Program.

Patient name	Optional. Enter the last, first, and middle name of the patient.

Gender	Required. Check Female, Male, or Other to indicate the gender of the patient.

Date of Birth	Required. Use this format: MM/DD/YYYY.

Ethnicity	Optional.

Race	Optional.

Event type	Required. BSI.

Date of event	Required to meet the BSI criterion occurred for the first time, during the Infection Window Period, using this format: MM/DD/YYYY.

**Table 2:** Summary of CLABSI predictors.

List of predictions:
(1) Multiple insertion.
(2) Site of insertion (femoral, subclavian, jugular).
(3) Rewire (Yes or No).
(4) Status of Procedure (Elective or Emergency).
(5) Co-morbidities (drop down selections):
(a) DM (Diabetes Mellitus),
(b) HTN (Hypertension),
(c) BA (Bronchial Asthma),
(d) Malignancy,
(e) Heart Disease etc.
(6) Compliant to Central Line Insertion & Maintenance bundle (Yes or No).
(7) Length of stay (*›*14 days).
(8) Parenteral nutrition (Yes or No).
(9) Antimicrobial usage (Yes or No).
(10) Renal failure (Yes or No).
(11) Surgical procedure (Yes or No).
(12) Age.
(13) Sex (Male or Female).
(14) Duration of device use.
(15) Transfer from other hospital (Yes or No).
(16) Transfer from other unit within the hospital (Yes or No).
(17) Co-existing infection (Yes or No).
(18) Temperature: (on admission & 48 hrs. after insertion).
(19) APACHE Score: 0-14(MILD); 15-29(MODERATE); ≥30 (SEVERE).
(20) Ventilated (Yes or No).
In addition, for neonates & children.
(21) Birth weight (≤750gm; 750-1000gm; 1001-1500gm; 1501-2500gm; *›*2500gm).
(22) Nasal CPAP (Yes or No).
(23) Blood Transfusion (Yes or No).
(24) Trauma (Yes or No).

**Table 3:** CLABSI indicators due to total symptom weightings.

Patient case Category	Symptoms weight	Type of Risk	Color Indicator
A	If sum of High symptoms	high risk	Red
	Criteria weights >=5		
B	If sum of Mild symptoms	mild risk	Orange
	Criteria weights >=5		
C	If sum of low symptoms	low risk	Yellow
	Criteria weights >=5		
D	If sum of symptoms	no risk	Green
	Criteria weights < 5		

**Table 4:** CLABSI symptoms used criteria for CLABSI infection prediction.

**No**	**PRD_NAME**	**VALUE_LABLE**	**VALUE_LEVEL**	**No**	**PRD_NAME**	**VALUE_LABLE**	**VALUE_LEVEL**
1	Site of insertion	No Risk	No Risk	37	Age	Premature	High Risk

2	Site of insertion	subclavian	Low Risk	38	Surgical procedure	No Risk	No Risk

3	Site of insertion	jugular	Mild Risk	39	Surgical procedure	Yes	High Risk

4	Site of insertion	femoral	High Risk	40	Sex	No Risk	No Risk

5	Rewire	No Risk	No Risk	41	Sex	Female	Low Risk

6	Rewire	Yes	High Risk	42	Sex	Male	High Risk

7	Status of Procedure	No Risk	No Risk	43	Duration of device use	No Risk	No Risk

8	Status of Procedure	Elective	Low Risk	44	Duration of device use	1 to 5 days	Low Risk

9	Status of Procedure	Emergency	High Risk	45	Duration of device use	6 to 10 days	Mild Risk

10	Co-morbidities	No Risk	No Risk	46	Duration of device use	More than 10 days	High Risk

11	Co-morbidities	Sickle Cell Anemia	High Risk	47	Transfer from other hospital	No Risk	No Risk

12	Co-morbidities	BA(Bronchial Asthma)	High Risk	48	Transfer from other hospital	Yes	High Risk

13	Co-morbidities	Meningitis	High Risk	49	Transfer from other unit within the hospital	No Risk	No Risk

14	Co-morbidities	Pulmonary Tuberculosis	High Risk	50	Transfer from other unit within the hospital	Yes	High Risk

15	Co-morbidities	Myocardial Infacrtion	High Risk	51	Co-existing infection	No Risk	No Risk

16	Co-morbidities	Chronic Liver Disease	High Risk	52	Co-existing infection	Yes	High Risk

17	Co-morbidities	Malignancy	High Risk	53	Temperature	No Risk	No Risk

18	Co-morbidities	HTN(Hypertension)	High Risk	54	Temperature	Yes	High Risk

19	Co-morbidities	DM(Diabetes Mellitus)	High Risk	55	APACHE Score	No Risk	No Risk

20	Co-morbidities	Cerebral Infarction	High Risk	56	APACHE Score	0 to 14	Low Risk

21	Co-morbidities	Heart Disease	High Risk	57	APACHE Score	15-29	Mild Risk

22	Compliant to Central Line Insertion & Maintenance bundle	Yes	No Risk	58	APACHE Score	>= 30	High Risk

23	Compliant to Central Line Insertion & Maintenance bundle	No	High Risk	59	Ventilated	No Risk	No Risk

24	Length of Stay	No Risk	No Risk	60	Ventilated	Yes	High Risk

25	Length of Stay	Less than equal 14 days	Mild Risk	61	Birth weight	No Risk	No Risk

26	Length of Stay	More than 14 days	High Risk	62	Birth weight	>2500	Low Risk

27	Parenteral nutrition	No Risk	No Risk	63	Birth weight	1000-1500	Mild Risk

28	Parenteral nutrition	Yes	High Risk	64	Birth weight	1501-2500	Mild Risk

29	Antimicrobial usage	No Risk	No Risk	65	Birth weight	<= 750 gm	High Risk

30	Antimicrobial usage	Yes	High Risk	66	Birth weight	750-1000 gm	High Risk

31	Renal failure	No Risk	No Risk	67	Nasal CPAP	No Risk	No Risk

32	Renal failure	Yes	High Risk	68	Nasal CPAP	Yes	High Risk

33	Age	No Risk	No Risk	69	Blood Transfusion	No Risk	No Risk

34	Age	Adult	Low Risk	70	Blood Transfusion	Yes	High Risk

35	Age	New Born	Mild Risk	71	Trauma	No Risk	No Risk

36	Age	Elderly	High Risk	72	Trauma	Yes	High Risk

**Table 5:** Percentage of the several categories of CLABSI risk.

Percentage of Risk of CLABSI for Each Group					
**MRN**	**High**	**Mild**	**Low**	**Normal**	**Final**
900088	***12/24=50%***	2/24=8.44%	1/24=4.17%	9/24=37.5%	High

900096	4/24=16.67%	1/24=4.17%	***5/24=20.83%***	14/24=58.33%	Low

900100	4/24=16.67%	***5/24=20.83%***	2/24=8.44%	13/24=54.17%	Mild

900124	4/24=16.67%	4/24=16.67%	3/24=12.5%	**13/24=54.17%**	Normal

900203	***9/24=37.5%***	1/24=4.17%	3/24=12.5%	11/24=45.83%	High

900593	***16/24=66.67%***	1/24=4.17%	2/24=8.44%	5/24=20.83%	High

900653	***12/24=50%***	2/24=8.44%	1/24=4.17%	9/24=37.5%	High

900753	***11/24=45.83%***	3/24=12.5%	3/24=12.5%	7/24=29.17%	High

## Data Availability

The data used to support the findings of this study are available from the corresponding author upon request.
